# Supplementary material for: Sperm Selection and Embryo Development: A Comparison of the Density Gradient Centrifugation and Microfluidic Chip Sperm Preparation Methods in Patients with Astheno-Teratozoospermia
Source: Life (Basel). 2021 Sep 7;11(9):933. doi: 10.3390/life11090933 (PMC8466221; doi:10.3390/life11090933)
Supplement: Supplementary file 1 [file life-11-00933-s001.zip › life-1356858-supplementary/Supplementary Table.pdf]

life

Supplementary Materials

Sperm Selection and Embryo Development: A Comparison of the Density Gradient Centrifugation and Microfluidic Chip Sperm Preparation Methods in Patients with Astheno-Teratozoospermia

Cagla Guler [1,2], Sureyya Melil [2], Umit Ozekici [2,3], Yaprak Donmez Cakil [4], Belgin Selam [5,*] and Mehmet Cincik [1,4]

Table S1. Baseline characteristics of the patients (mean ± SD)

Age	BMI (kg/m²)	Induction days	Induction dose (mg)
32.55±4.68<br>(23–38)	24.05±4.89<br>(19.3–36.8)	9.91±1.41<br>(7–13)	2952.27±447.34<br>(2100–3900)

Table S2. The number of sibling oocytes

Patient	Number of oocytes undergoing ICSI	Number of sibling oocytes	
		Microchip group	Gradient group
**1**	6	3	3
2	3	2	1
3	12	5	5
4	4	2	2
5	10	5	5
6	9	4	5
7	14	7	7
8	5	3	2
9	9	4	5
10	7	4	3
11	5	3	2
12	5	3	2
13	23	12	11
14	9	5	4
15	17	9	8
16	6	3	3
17	10	5	5
18	9	4	5
19	4	2	2
20	13	7	6
21	11	6	5
22	12	6	6
