# Supplementary figures and images for: Sperm Selection and Embryo Development: A Comparison of the Density Gradient Centrifugation and Microfluidic Chip Sperm Preparation Methods in Patients with Astheno-Teratozoospermia
Source: Life (Basel). 2021 Sep 7;11(9):933. doi: 10.3390/life11090933 (PMC8466221; doi:10.3390/life11090933)

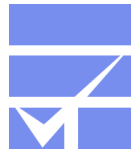

# CONSORT

TRANSPARENT REPORTING of TRIALS

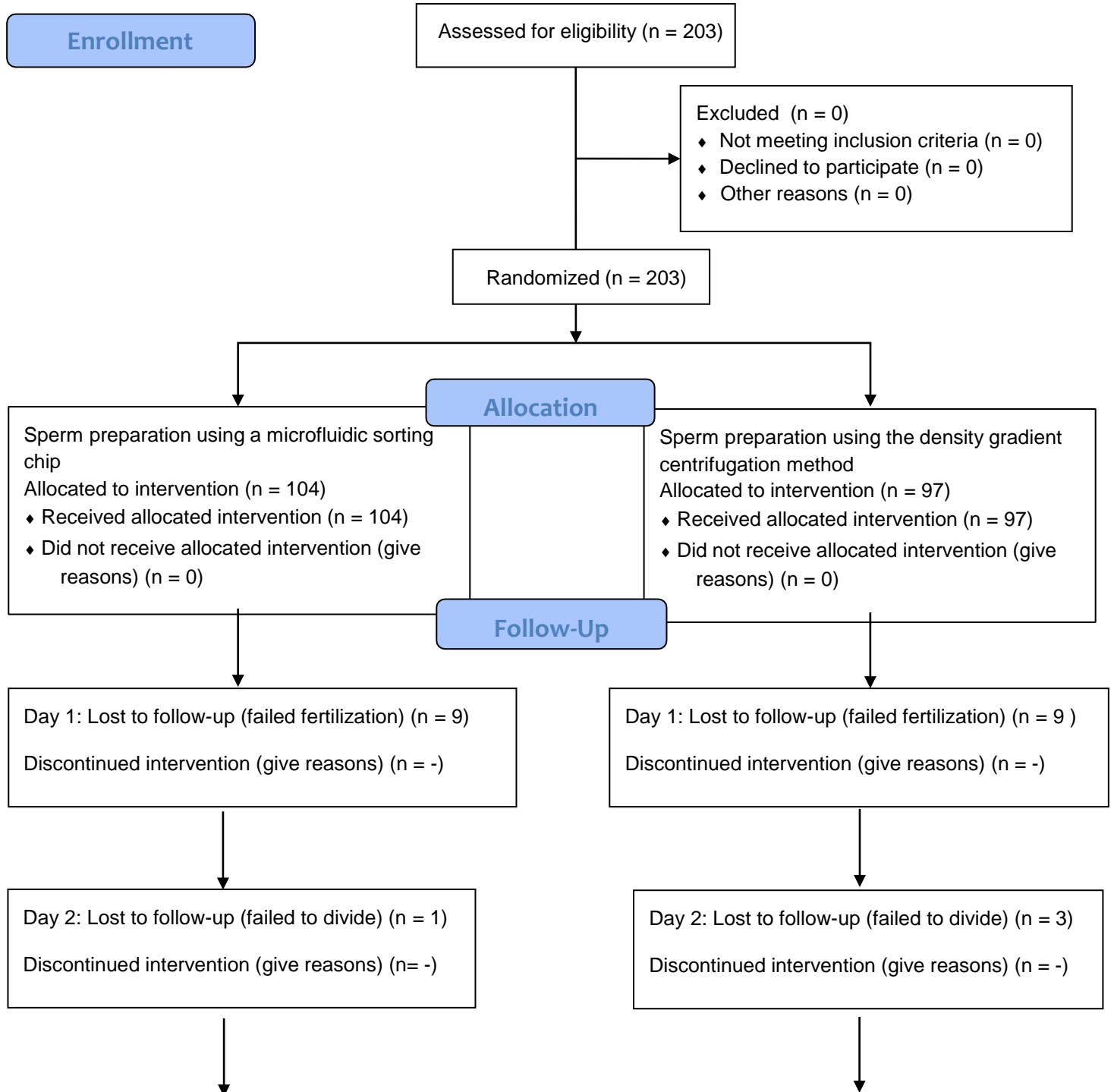

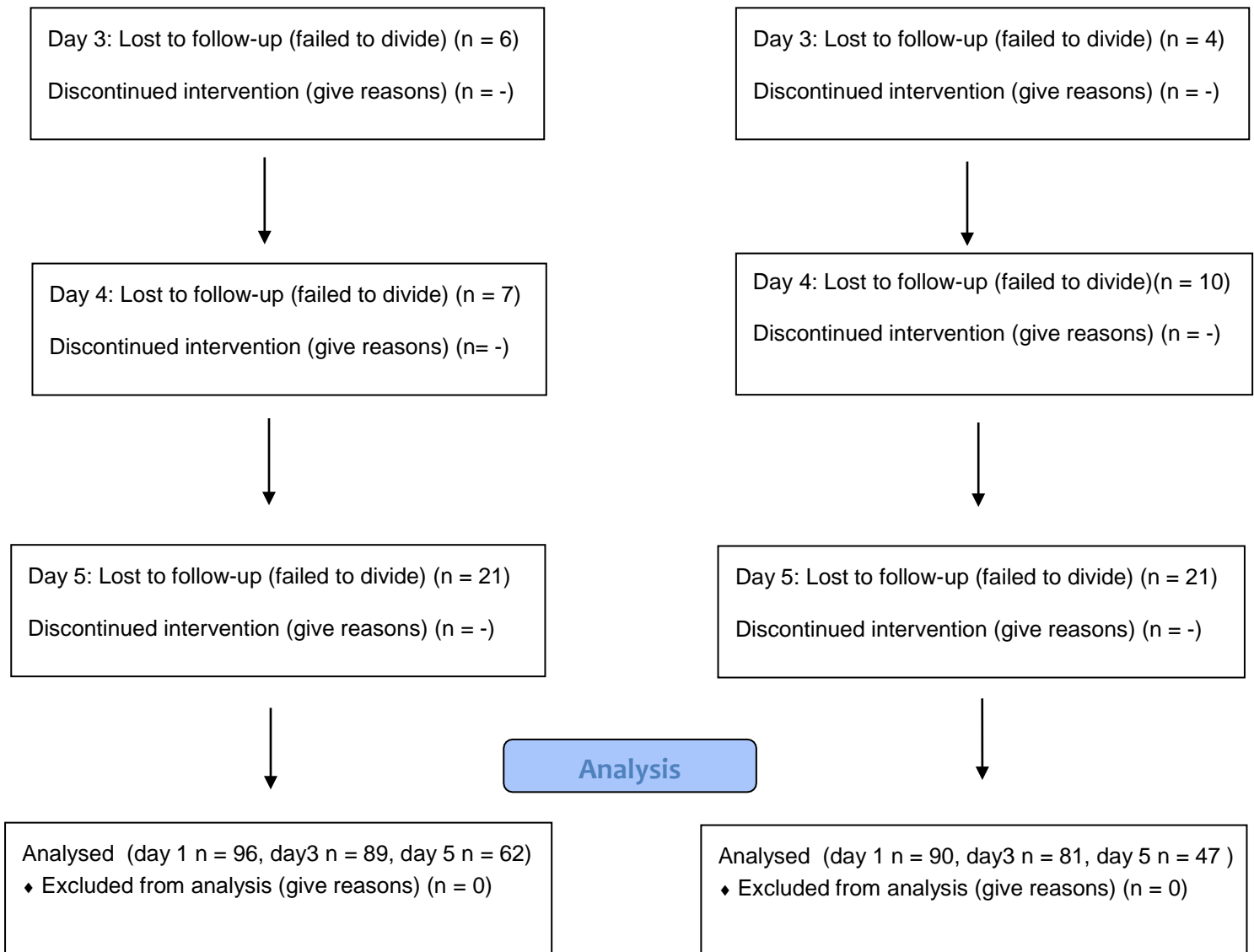

Figure S1. CONSORT 2010 Flow Diagram.

Supplement: Supplementary file 1 [file life-11-00933-s001.zip › life-1356858-supplementary/Supplementary Figure 1.pdf]
